# Research on the economic loss of hospital-acquired pneumonia caused by Klebsiella pneumonia base on propensity score matching

**DOI:** 10.1097/MD.0000000000025440

**Published:** 2021-04-16

**Authors:** Xiao Zhong, Dong-Li Wang, Li-Hua Xiao

**Affiliations:** a Department of Nosocomial Infection, University of Chinese Academy of Sciences, Shenzhen Hospital; b Inspection Center, Guangming District Center for Disease Control and Prevention, Shenzhen, Guangdong, China.

**Keywords:** direct economic loss, extended-spectrum beta-lactamases, hospital-acquired pneumonia, Klebsiella pneumonia, length of stay, propensity score matching

## Abstract

**Background:**

Hospital-acquired pneumonia (HAP) caused by Klebsiella pneumonia (KP) is a common nosocomial infection (NI). However, the reports on the economic burden of hospital-acquired pneumonia caused by Klebsiella pneumonia (KP-HAP) were scarce. The study aims to study the direct economic loss caused by KP-HAP with the method of propensity score matching (PSM) to provide a basis for the cost accounting of NI and provide references for the formulation of infection control measures.

**Methods:**

A retrospective investigation was conducted on the hospitalization information of all patients discharged from a tertiary group hospital in Shenzhen, Guangdong province, China, from June 2016 to August 2019. According to the inclusion and exclusion criteria, patients were divided into the HAP group and noninfection group, the extended-spectrum beta-lactamases (ESBLs) positive KP infection group, and the ESBLs-negative KP infection group. After the baselines of each group were balanced with the PSM, length of stay (LOS) and hospital cost of each group were compared.

**Results:**

After the PSM, there were no differences in the baselines of each group. Compared with the noninfection group, the median LOS in the KP-HAP group increased by 15 days (2.14 times), and the median hospital costs increased by 7329 yuan (0.89 times). Compared with the ESBLs-negative KP-HAP group, the median LOS in the ESBLs-positive KP-HAP group increased by 7.5 days (0.39 times), and the median hospital costs increased by 22,424 yuan (1.90 times).

**Conclusion:**

KP-HAP prolonged LOS and increased hospital costs, and HAP caused by ESBLs-positive KP had more economic losses than ESBLs-negative, which deserves our attention and should be controlled by practical measures.

## 1 Introduction

Nosocomial infectious pneumonia (NP) includes ventilator-associated pneumonia (VAP), hospital-acquired pneumonia (HAP), and healthcare-associated pneumonia (HCAP).^[1]^ It is the most common nosocomial infection (NI) except urinary tract infection,^[2]^ and usually the second most common cause of NIs overseas.^[3,4]^ In the United States, NP accounted for 21.8% of NIs, and 60.9% of NP were HAP.^[5]^ In China, it accounted for 47.2%, which was the most significant proportion of NIs, and 92.3% of NP were HAP.^[6]^ NP could result in high mortality,^[7–9]^ which accounted for the leading cause of death from all NIs,^[10,11]^ prolonged length of stay (LOS), increased hospital costs for patients.^[12]^

At present, China's medical service payment method is a mixed payment system. It can be divided into 2 categories: self-paying medical treatment and medical insurance. Medical insurance includes medical social insurance and commercial medical insurance. Medical social insurance is divided into 3 kinds: urban employees’ basic medical insurance (UEBMI), urban residents’ basic medical insurance (URBMI), and the new rural cooperative medical system (NRCMS). In most areas of China, the medical insurance compensation system, according to service items, is still implemented, and the extra costs caused by NIs are paid by patients. The impact of NIs on the hospital economy is only reflected in the reduction of bed turnover rate. Under this kind of medical payment method, it is easy to cause unnecessary medical treatments, which is not conducive to reducing the medical expenses of patients. Starting from the end of 2017, the government will gradually implement the prepayment system for single diseases.^[13]^ Under this single disease medical insurance, the hospital will bear the part of the hospital expenses beyond the pre-payment due to the NIs. However, for patients, it can significantly reduce the cost of hospitalization. Therefore, it is necessary to study the economic losses caused by NIs, to attract the attention of the hospitals and try to control the occurrence of NIs.

Some studies had shown that KP was a common pathogenic bacteria of HAP, accounting for about 8.9% to 12%.^[14–16]^ The study of the economic loss of hospital-acquired pneumonia caused by Klebsiella pneumonia (KP-HAP) can provide references for the cost accounting of NIs, the establishment and effect evaluation of NIs prevention and control measures. There had been many reports on the economic losses caused by VAP.^[17–19]^ However, the reports on the HAP, especially KP-HAP, were scarce. Therefore, we aimed to calculate the direct economic losses and the extension of LOS of KP-HAP, provide references for its cost accounting.

## 2 Patients and methods

### 2.1 Study population and setting

This study was conducted in a tertiary group hospital in Guangming District, Shenzhen, Guangdong province, China, which has 2 hospitals: 887 beds in the west hospital and 463 beds in the east hospital, with an average annual inpatient population of 80,000. All patients discharged from June 2016 to August 2019 were selected as the study subjects. The study was approved by the Ethics Committee of the Shenzhen Hospital of the University of Chinese Academy of Sciences, and all the information of patients was kept confidential in the study.

### 2.2 Inclusion and exclusion criteria

The case group included patients with HAP caused by KP, excluding patients on a ventilator and patients infected with a mixture of pathogenic bacteria. The control group included all patients discharged during the study period, excluding ventilator users, and infection patients. In the case and control group, the patients discharged due to death, the absence of important information such as demographic information, hospitalization information, and diagnostic information were also excluded.

### 2.3 HAP diagnosis

According to the ministry of health of the People's Republic of China “hospital infection diagnosis standard 2001,”^[20]^ HAP is defined as the lower respiratory infection that develops in a hospitalized patient after 48 hours of admission, and was not present or incubating at the time of admission. First, when the clinicians discover the HAP, they can report it to the infection control department through the NI information system. Then, 2 experienced hospital infection professionals will judge whether it is an NI according to the standard. When the 2 people disagree, the third professional will make the final decision. Besides, the system will give an early warning of possible NI cases according to the patient's temperature, microbial culture, inflammatory indicators, and the use of antibiotics. The professionals of infection control will take the initiative to deal with the early warnings and judge whether they are NIs or not. Through this system, the inpatient information and NI information can be obtained timely and accurately.

### 2.4 Definition of outcome indicators

LOS = the day of discharge–the day of admission; Increment of LOS = median LOS in the case group-median LOS in the control group; Increment of hospital cost = median hospital cost in case group-median hospital cost in the control group.

### 2.5 Case and control selection and matching

The case and control groups were selected according to inclusion and exclusion criteria. Through the NI information system (NIIS), hospital information system (HIS), and medical record management system (MRMS), patients’ demographic data, diagnosis information encoded by ICD10, diagnosis and treatment-related information, LOS, and hospitalization expenses were collected. The patients were divided into the KP-HAP group (The group of patients with HAP caused by KP during admission), the noninfection group (The group of patients without infection during admission), the extended-spectrum beta-lactamases (ESBLs)-positive KP group (The group of patients with HAP caused by ESBLs-positive KP during admission), and the ESBLs-negative KP group (The group of patients with HAP caused by ESBLs-negative KP during admission).

The propensity score matching (PSM) method was first proposed by Rosenbaum^[21]^ in the 1980 s and is widely used in observational studies that need to control for more confounding bias. It is to use the propensity score to synthesize the information of all the observed variables to achieve the goal of balancing the variables and reducing the bias. Firstly, logistic regression was used to calculate the propensity score of each patient. Then the 1:1 case-control matching was conducted according to the principle of neighboring matching and caliper matching (caliper value: 0.05). The matching variables were the patients’ age, gender, primary diagnosis (classified by the ICD 10), whether or not surgery, intensive care unit (ICU) admission, whether indwelling central venous catheter, whether patients with urethral catheter, the complications associated with increased hospital costs^[22]^ (malignant tumor, heart failure, coronary heart disease, diabetes, hypertension, chronic obstructive pulmonary disease (COPD), dementia, depression, etc), Charlson comorbidity index, illness severity classification, whether the emergency, the terms of payment, and LOS before infection. After matching, the median LOS and median hospitalization costs in the 2 groups were compared. All patients’ costs were converted into 2019 costs based on the CPI index published on China's National Bureau of Statistics Website.^[23]^

In the comparison of KP-HAP with ESBLs-positive and ESBLs-negative, the baselines of 2 groups before matching the confounding variables had been able to achieve balance. One possible reason might be that the exposure factors listed were temporarily evenly distributed between the 2 groups due to the limited sample size. Or some of the exposure characteristics of the study variables such as antimicrobial use were not included due to data source constraints, which led to the baseline conditions identical. For avoiding the losses of samples, the 2 groups were directly compared instead of matching their propensity scores.

### 2.6 Culture techniques

Respiratory specimens were obtained following standard operating procedures by nurses. Samples not immediately cultured were refrigerated at 4°C. The identification of microorganisms and confirmatory tests to identify the ESBL producer followed the Clinical Laboratory Standards Institute (CLSI) recommends.

### 2.7 Cost estimation

The hospitalization expenses of the patients included the costs of general medical service, general treatment operation, nursing care, pathological diagnosis, laboratory diagnosis, imaging diagnosis, clinical diagnosis, nonsurgical treatment, surgery, rehabilitation, medicine, and disposable medical supplies. All information was obtained from the HIS.

### 2.8 Statistic analysis

The data were inputted into Excel 2016 to establish the database, and Stata 15.1 (Stata Corporation, College Station, TX, US) software was used for PSM and other statistical analysis. The continuous variables accord with normal distribution were described by the means with standard deviation (SD), and non-normal distribution data were defined by the medians with interquartile range (IQR). The difference between groups was compared with the *t* test or Wilcoxon rank test. The categorical variables were described by frequencies and percentages, and the comparisons between the groups were conducted by a Chi-Squared test or Fisher exact probability method. Statistical significance was observed at an *α* level of 0.05.

## 3 Results

### 3.1 The general information of this study

**Figure 1:**
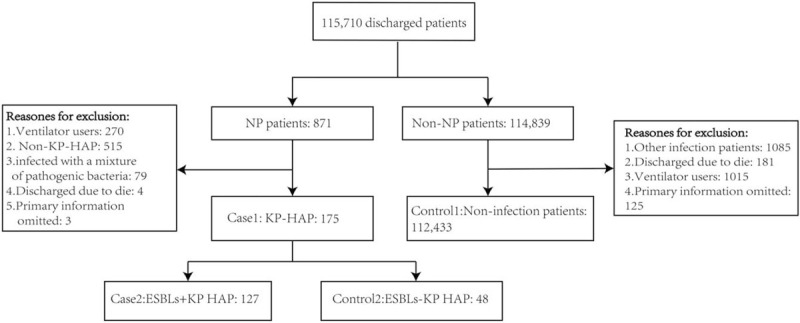
Summary of the inclusion and exclusion of the case and control groups. NP = nosocomial infectious pneumonia; HAP = hospital-acquired pneumonia; ESBLs = extended-spectrum beta-lactamases; KP-HAP = hospital-acquired pneumonia caused by Klebsiella pneumonia; ESBLs+ = ESBLs positive; ESBLs- = ESBLs negative.

From June 2016 to August 2019, a total of 115,710 patients were discharged from the hospital, with 871 NP cases and an incidence of 0.75%. After some patients were excluded according to the inclusion and exclusion criteria, 175 patients of KP-HAP were included in the case group, including 127 patients of ESBLs-negative KP and 48 patients of ESBLs-positive KP, and the control group with noninfection had 112,433 patients, as shown in Figure 1.

### 3.2 Comparisons of the baseline conditions between the KP-HAP and noninfection groups before PSM

Before PSM, baseline conditions of the KP-HAP group and the noninfection group were unbalanced. In the KP-HAP group, most of the patients were male, and the age, Charlson index, and severity of illness were all larger than those of the control group. The increments of median LOS in patients with KP-HAP were 15 days (3 times), and the increments of median hospital expenses were 9974.76 yuan (1.79 times), as shown in Table 1.

**Table 1:** Baseline characteristics of hospital-acquired pneumonia caused by Klebsiella pneumonia and noninfection groups before matching.

Characteristics	KP-HAP (n = 175)	Noninfection (n = 112433)	*χ*^*2*^ or Z	*P* value
Male sex, n (%)	114 (65.14)	53920 (47.96)	20.675	<.001
Admission to ICU, n (%)	44 (25.14)	1334 (1.19)		<.001
CVC, n (%)	47 (26.86)	2719 (2.42)		<.001
UC, n (%)	85 (48.57)	13891 (12.35)	210.821	<.001
Principal diagnosis				<.001
Infectious and parasitic diseases, n (%)	0	4031 (3.59)		
Neoplasms, n (%)	16 (9.14)	4872 (4.33)		
Blood and blood-forming organs, n (%)	2 (1.14)	759 (.68)		
Endocrine, nutritional, and metabolic, n (%)	3 (1.71)	2492 (2.22)		
Mental and behavioural disorders, n (%)	0	456 (.41)		
The nervous system, n (%)	7 (4.00)	2331 (2.07)		
Eye and adnexa, n (%)	0	427 (.38)		
Ear and mastoid process, n (%)	0	1327 (1.18)		
Circulatory system, n (%)	68 (38.86)	11516 (10.24)		
Respiratory system, n (%)	4 (2.29)	15751 (14.01)		
Digestive system, n (%)	12 (6.86)	11113 (9.88)		
Skin and subcutaneous tissue, n (%)	1 (.57)	919 (.82)		
Musculoskeletal and connective tissue, n (%)	4 (2.29)	5524 (4.91)		
The genitourinary system, n (%)	8 (4.57)	8145 (7.24)		
Pregnancy, childbirth, and puerperium, n (%)	11 (6.29)	22034 (19.60)		
Certain conditions in the perinatal, n (%)	5 (2.86)	4621 (4.11)		
Congenital malformations., n (%)	0	996 (.89)		
Symptoms. not elsewhere classified, n (%)	0	569 (.51)		
Injury, poisoning, n (%)	32 (18.29)	10780 (9.59)		
Factors influencing health status, n (%)	2 (1.14)	3770 (3.35)		
Comorbidities				
Diabetes, n (%)	24 (13.71)	7960 (7.08)	11.675	.001
Cancer, n (%)	16 (9.14)	3127 (2.78)		<.001
Stroke, n (%)	34 (19.43)	3894 (3.46)	132.290	<.001
Asthma, n (%)	3 (1.71)	1363 (1.21)		.48
Congestive heart failure, n (%)	8 (4.57)	988 (.88)		<.001
Ischemic heart disease, n (%)	18 (10.29)	3425 (3.05)	30.895	<.001
Hypertension, n (%)	60 (34.29)	12104 (10.77)	100.319	<.001
COPD, n (%)	6 (3.43)	1160 (1.03)		.01
Dementia, n (%)	2 (1.14)	55 (.05)		.004
Depression, n (%)	2 (1.14)	134 (.12)		.02
Severity classification^∗^			504.451	<.001
A, n (%)	11 (6.29)	17786 (15.82)		
B, n (%)	25 (14.29)	37048 (32.95)		
C, n (%)	58 (33.14)	50881 (45.25)		
D, n (%)	81 (46.29)	6718 (5.98)		
Surgery, n (%)	130 (74.29)	59621 (53.03)	31.702	<.001
Emergency, n (%)	87 (49.71)	78080 (69.45)	32.042	<.001
Terms of payment				.70
UEBMI, n (%)	0	173 (.15)		
URBMI, n (%)	76 (43.43)	52976 (47.12)		
NRCMS, n (%)	0	8 (.01)		
Impoverished rescue, n (%)	0	4 (.00)		
Commercial health insurance, n (%)	0	2 (.00)		
Free medicare, n (%)	0	1 (.00)		
Self-paying, n (%)	91 (52.00)	54523 (48.49)		
Others, n (%)	8 (4.57)	4746 (4.22)		
Age in yr ± SD	50.98 ± 1.60	33.44 ± 0.06	12.131^∗∗^	<.001
LOS before the infection, in d, median (IQR)	5 (3, 8)	5 (3, 7)	2.913^#^	.004
Charson index, median (IQR)	3 (1, 5)	0 (0, 2)	11.630^#^	<.001
LOS, in d, median (IQR)	20 (13, 29)	5 (4, 8)	19.151^#^	<.001
Total cost, median (IQR)	15542 (9938,28372)	5567.24 (3383, 10129)	8.304^#^	<.001

### 3.3 Comparisons between the KP-HAP and noninfection groups after PSM

After PSM, 175 cases in the case group were successfully matched, and the distributions of covariates in the KP-HAP group and the noninfection group were balanced. Compared with the control group, the median LOS in the KP-HAP group increased by 15 days (2.14 times), and the median hospital cost increased by 7329 yuan (0.89 times), as shown in the Table 2.

**Table 2:** Comparisons between hospital-acquired pneumonia caused by Klebsiella pneumonia and noninfection groups after propensity score matching.

Characteristics	KP-HAP (n = 175)	Noninfection (n = 175)	*χ*^*2*^ or Z	*P* value
Male sex, n (%)	114 (65.14)	130 (74.29)	3.464	.06
Admission to ICU, n (%)	44 (25.14)	41 (23.43)	.140	.71
CVC, n (%)	47 (26.86)	44 (25.14)	.134	.72
UC, n (%)	85 (48.57)	81 (46.29)	.183	.67
Principal diagnosis				.08
Infectious and parasitic diseases, n (%)	0	5 (2.86)		
Neoplasms, n (%)	16 (9.14)	12 (6.86)		
Blood and blood-forming organs, n (%)	2 (1.14)	1 (.57)		
Endocrine, nutritional, and metabolic, n (%)	3 (1.71)	5 (2.86)		
Mental and behavioral disorders, n (%)	0	1 (.57)		
The nervous system, n (%)	7 (4.00)	9 (5.14)		
Eye and adnexa, n (%)	0	1 (.57)		
Ear and mastoid process, n (%)	0	2 (1.14)		
Circulatory system, n (%)	68 (38.86)	45 (25.71)		
Respiratory system, n (%)	4 (2.29)	11 (6.29)		
Digestive system, n (%)	12 (6.86)	16 (9.14)		
Skin and subcutaneous tissue, n (%)	1 (.57)	1 (.57)		
Musculoskeletal and connective tissue, n (%)	4 (2.29)	3 (1.71)		
The genitourinary system, n (%)	8 (4.57)	8 (4.57)		
Pregnancy, childbirth and puerperium, n (%)	11 (6.29)	14 (8.00)		
Certain conditions in the perinatal, n (%)	5 (2.86)	2 (1.14)		
Symptoms. not elsewhere classified, n (%)	0	3 (1.71)		
Congenital malformations, n (%)	0	0		
Injury, poisoning, n (%)	32 (18.29)	30 (17.14)		
Factors influencing health status, n (%)	2 (1.14)	6 (3.43)		
Comorbidities				
Diabetes, n (%)	24 (13.71)	26 (14.86)	.093	.76
Cancer, n (%)	16 (9.14)	22 (12.57)	1.063	.30
Stroke, n (%)	34 (19.43)	36 (20.57)	.071	.79
Asthma, n (%)	3 (1.71)	4 (2.29)		1.00
Congestive heart failure, n (%)	8 (4.57)	7 (4.00)	.070	.79
Ischemic heart disease, n (%)	18 (10.29)	18 (10.29)	.000	1.00
Hypertension, n (%)	60 (34.29)	49 (28.00)	1.612	.20
COPD, n (%)	6 (3.43)	9 (5.14)	.627	.43
Dementia, n (%)	2 (1.14)	6 (3.43)		.28
Depression, n (%)	2 (1.14)	1 (0.57)		1.00
Severity classification^∗^			6.425	.09
A, n (%)	11 (6.29)	10 (5.71)		
B, n (%)	25 (14.29)	19 (10.86)		
C, n (%)	58 (33.14)	81 (46.29)		
D, n (%)	81 (46.29)	65 (37.14)		
Surgery, n (%)	130 (74.29)	128 (73.14)	.059	.81
Emergency, n (%)	87 (49.71)	96 (54.86)	.928	.34
Terms of payment			.579	.75
URBMI, n (%)	76 (43.43)	83 (47.43)		
Self-paying, n (%)	91 (52.00)	85 (48.57)		
Others, n (%)	8 (4.57)	7 (4.00)		
Age in yr ± SD	50.98 ± 1.60	52.24 ± 1.55	.565^∗∗^	.57
LOS before the infection, in d, median (IQR)	5 (3, 8)	5 (4, 8)	.664^#^	.51
Charson index, median (IQR)	3 (1, 5)	3 (1, 6)	1.015^#^	.31
LOS, in days, median (IQR)	22 (14, 29)	7 (4, 12)	−11.160^#^	<.001
Total cost, median (IQR)	15542 (9938, 28372)	8213 (4610, 15013)	−7.536^#^	<.001

### 3.4 Comparisons between the ESBLs-positive KP-HAP and ESBLs-negative KP-HAP groups

In 48 patients with ESBLs-positive KP-HAP and 127 patients with ESBLs-negative KP-HAP, the baseline conditions between the 2 groups were balanced. Compared with the ESBLs-negative KP-HAP group, the median LOS in the ESBLs-positive KP-HAP group increased by 7.5 days, 0.39 times, and the median hospital cost increased by 22,424 yuan, 1.90 times, as shown in Table 3.

**Table 3:** Comparisons between hospital-acquired pneumonia caused by extended-spectrum beta-lactamases producing Klebsiella pneumonia and hospital-acquired pneumonia caused by extended-spectrum beta-lactamases negative Klebsiella pneumonia.

Characteristics	ESBLs + KP HAP (n = 48)	ESBLs-KP HAP (n = 127)	*χ*^*2*^ or Z	*P* value
Male sex, n (%)	30 (62.50)	84 (66.14)	.204	.65
Admission to ICU, n (%)	13 (27.08)	31 (24.41)	.132	.72
CVC, n (%)	13 (27.08)	34 (26.77)	.002	.97
UC, n (%)	24 (50.00)	61 (48.03)	.054	.82
Principal diagnosis				.13
Neoplasms, n (%)	4 (8.33)	12 (9.45)		
Blood and blood-forming organs, n (%)	1 (2.08)	1 (.79)		
Endocrine, nutritional and metabolic, n (%)	1 (2.08)	2 (1.57)		
The nervous system, n (%)	4 (8.33)	3 (2.36)		
Circulatory system, n (%)	11 (22.92)	57 (44.88)		
Respiratory system, n (%)	2 (4.17)	2 (1.57)		
Digestive system, n (%)	5 (10.42)	7 (5.51)		
Skin and subcutaneous tissue, n (%)	1 (2.08)	0		
Musculoskeletal and connective tissue, n (%)	1 (2.08)	3 (2.36)		
The genitourinary system, n (%)	1 (2.08)	7 (5.51)		
Pregnancy, childbirth and puerperium, n (%)	4 (8.33)	7 (5.51)		
Certain conditions in the perinatal, n (%)	1 (2.08)	4 (3.15)		
Injury, poisoning, n (%)	11 (22.92)	21 (16.54)		
Factors influencing health status, n (%)	1 (2.08)	1 (.79)		
Comorbidities				
Diabetes, n (%)	8 (16.67)	16 (12.60)	.487	.49
Cancer, n (%)	6 (12.50)	10 (7.87)	.897	.34
Stroke, n (%)	8 (16.67)	26 (20.47)	.322	.57
Asthma, n (%)	0	3 (2.36)		.56
Congestive heart failure, n (%)	2 (4.17)	6 (4.72)		1.00
Ischemic heart disease, n (%)	4 (8.33)	14 (11.02)		.78
Hypertension, n (%)	18 (37.50)	42 (33.07)	.303	.58
COPD, n (%)	2 (4.17)	4 (3.15)		.67
Dementia, n (%)	0	2 (1.57)		1.00
Depression, n (%)	1 (2.08)	1 (0.79)		.47
Severity classification^∗^				.80
A, n (%)	3 (6.25)	8 (6.30)		
B, n (%)	5 (10.42)	20 (15.75)		
C, n (%)	18 (37.50)	40 (31.50)		
D, n (%)	22 (45.83)	59 (46.46)		
Surgery, n (%)	38 (79.17)	92 (72.44)	.825	.36
Emergency, n (%)	25 (52.08)	62 (48.82)	.149	.70
Terms of payment				.92
URBMI, n (%)	22 (45.83)	54 (42.52)		
Self-paying, n (%)	24 (50.00)	67 (52.76)		
Others, n (%)	2 (4.17)	6 (4.72)		
Age in yr ± SD	51.17 ± 3.08	50.91 ± 1.88	−.072^∗^	.94
LOS before the infection, in days, median (IQR)	5 (3, 8)	5 (4, 8)	.172^#^	.86
Charson index, median (IQR)	2.5 (0, 5)	3 (1, 5)	−.157^#^	.88
LOS, in days, median (IQR)	26.5 (19, 29)	19 (12, 29)	−3.020^#^	.003
Total cost, median (IQR)	34218 (27096, 45691.5)	11794 (9026, 17183)	−8.597^#^	<.001

## 4 Discussion

In traditional NIs economic loss studies, the additional lengths and costs of hospital stay due to NIs were calculated from the time the patient was admitted to the hospital, not from the time the infection occurred.^[24]^ Besides, patients with short hospital stay were easily selected into the control group,^[25]^ which led to the generation of time-dependent bias and exaggerated the study outcomes. In this study, HAP patients’ LOS before infection were matched with the LOS in the control group, and only those patients whose LOS were longer than or equal to the case group were selected as the control group, which could minimize the influence of time dependence bias. Moreover, in the case-control study of the health economics of NIs, the critical factors that affected the hospitalization cost mainly included the principal diagnosis, potential comorbidities, and severity of the patient's condition, etc. Therefore, we matched the primary diagnosis, Charlson index, and severity classification of the disease to avoid their influence on the study.

Research on the economic costs of VAP has been widely reported, showing that VAP increased hospitalization costs by $10,000 to $40,000.^[17–19]^ The economic loss of VAP was severe, but the total economic loss of HAP was more severe than VAP due to its higher incidence.^[5]^ Therefore, this study focused on the economic loss of HAP, which was more common than VAP. It was noticed that many studies on the economic loss of NP did not distinguish HAP and VAP. For example, Victor d. Rosenthal et al used a matching method to study ICU acquired pneumonia, which extended the LOS by 8.95 days and increased the cost of hospitalization by $2238.^[26]^ A Chinese case-control matching study showed that NP caused an economic loss of 31,940 yuan and prolonged LOS of 34.39 days.^[27]^ However, our study excluded patients on ventilators and determined the economic loss of HAP. In this study, compared with patients without NIs, patients with KP-HAP had a 15 days LOS extension (2.14 times), and the median hospital cost increased by 7329 yuan (0.89 times), indicating that HAP caused a severe economic burden for patients, which was consistent with the results of many studies.^[28,29]^

Moreover, ESBLs-positive KP-HAP increased hospital expenses by 22,424 yuan ($3200) compared with ESBLs-negative. A PSM study in a hospital of the same level in Sichuan, China^[30]^ showed that: carbapenems antimicrobial-resistant KP-HAP had an economic loss of $10,192 more than carbapenems antimicrobial sensitive, which was much higher than our results. It is suggested that carbapenems resistant KP infections might cause more severe economic losses. Furthermore, we found that the economic losses of ESBLs-positive infections were more severe than that of ESBLs-negative infections. It was consistent with a study of ESBLs-positive *Escherichia coli* bloodstream infections.^[31]^

After the literature search, no references were found on the economic losses of KP-HAP. The outcomes of some studies on the economic losses of HAP were different from our research. For example, Flanders SA et al reported that the HAP prolonged the LOS for 7 to 10 days.^[2]^ Ott e. et al compared the economic loss of MRSA-HAP with MSSA-HAP (60,684 vs 38,731 dm, 0.57 times).^[32]^ In the study of Cakir Edis E et al, the bed cost of HAP was $4783.12 times more than that of nonpneumonia patients, and the LOS was increased by 23 days, 3.5 times.^[33]^ The discrepancy may be due to the differences in study subjects, including patients with VAP, study methods, pathogens, and regional economic development.

At present, many of the studies on the economic losses of NIs reported in domestic literature are descriptive studies matching a few variables such as age and gender, which may have a significant confounding bias. In this study, we used PSM and matched patients’ age, gender, primary diagnosis (classified by the ICD 10), whether or not surgery, ICU admission, whether indwelling central venous catheter, whether patients with the urethral catheter, the complications associated with increased hospital costs (cancer, heart failure, ischemic heart disease, diabetes, hypertension, COPD, dementia, depression), Charlson comorbidity index, illness severity classification, whether the emergency, the terms of payment, and LOS before infection, etc, a total of 20 variables. The influences of confounding factors were reduced as much as possible, and the comparability between the 2 groups was improved.

However, there were still deficiencies in the study: first, this study was a retrospective study, which inevitably led to retrospective bias. Second, although most variables were matched, there might still be unmatched variables that had not been considered, and there might also be other confounding bias. Finally, the hospital charge in this study was based on the charge standard of the region and our hospital so that it may be different from the results of similar studies in other areas or other hospitals.

## 5 Conclusions

Summarily, KP-HAP not only lengthened the LOS but also significantly increased the hospital costs, and ESBLs-positive KP-HAP had more economic losses than the ESBLs-negative, which should arouse the concerns of the hospital administrators and develop targeted and practical measures for prevention and control.

## Acknowledgments

The authors would like to thank the professionals of the department of infection control and the microbiology department at the University of Chinese academy of sciences Shenzhen hospital for their hard work.

## Author contributions

**Conceptualization:** Xiao Zhong.

**Data curation:** Dong Li Wang.

**Investigation:** Dong Li Wang, Li Hua Xiao.

**Methodology:** Xiao Zhong.

**Supervision:** Li Hua Xiao.

**Writing – original draft:** Xiao Zhong.

**Writing – review & editing:** Xiao Zhong, Dong Li Wang.
